# Processive DNA synthesis is associated with localized decompaction of constitutive heterochromatin at the sites of DNA replication and repair

**DOI:** 10.1080/19491034.2019.1688932

**Published:** 2019-11-19

**Authors:** Vadim O. Chagin, Britta Reinhart, Annette Becker, Oliver Mortusewicz, K. Laurence Jost, Alexander Rapp, Heinrich Leonhardt, M. Cristina Cardoso

**Affiliations:** a Cell Biology & Epigenetics, Department of Biology, Technische Universität Darmstadt, Darmstadt, Germany; b Institute of Cytology, Russian Academy of Sciences, St. Petersburg, Russia; c Department of Biology II, LMU Munich, Munich, Germany

**Keywords:** DNA replication, DNA repair, pericentromeric heterochromatin, chromocenters, chromatin compaction, processive DNA synthesis, genome architecture, PCNA

## Abstract

Constitutive heterochromatin is considered as a functionally inert genome compartment, important for its architecture and stability. How such stable structure is maintained is not well understood. Here, we apply four different visualization schemes to label it and investigate its dynamics during DNA replication and repair. We show that replisomes assemble over the heterochromatin in a temporally ordered manner. Furthermore, heterochromatin undergoes transient decompaction locally at the active sites of DNA synthesis. Using selective laser microirradiation conditions that lead to damage repaired via processive DNA synthesis, we measured similarly local decompaction of heterochromatin. In both cases, we could not observe large-scale movement of heterochromatin to the domain surface. Instead, the processive DNA synthesis machinery assembled at the replication/repair sites. Altogether, our data are compatible with a progression of DNA replication/repair along the chromatin in a dynamic mode with localized and transient decompaction that does not globally remodels the whole heterochromatin compartment.

## Introduction

Chromatin represents genomic DNA that is hierarchically organized and compacted several hundred times within the interphase nucleus by interaction with proteins and RNAs. At the first level of chromatin organization, ~150 bp DNA segments are wrapped around octamers of core histone proteins to form the 11 nm nucleosome fiber [1]. The nucleosome fiber can be further compacted through interaction with linker histone H1 [2] and other chromatin architectural proteins [3]. Existence of a secondary order chromatin organization in the form of 30 nm fibers *in vivo* has been intensely debated and several alternative models of higher-order chromatin organization have been proposed to describe higher-order levels of genome organization within the interphase nuclear volume [4–9].

The functional status of chromatin is linked to the particular pattern of epigenetic signatures [10,11] and reflected in the level of its relative compaction. Hence, actively transcribed euchromatin regions are believed to exist in so-called ‘open’ chromatin conformation, while transcriptionally inert heterochromatin has the most compacted state [12].

Despite differences in their compaction level, all parts of the genome participate in such aspects of nuclear DNA metabolism as DNA replication and repair of DNA damage. Protein machineries synthetizing DNA in these nuclear processes must have access to the naked DNA strands, thus, requiring a complete dismantling of the chromatin architecture. Intuitively, the more compact structure of heterochromatin [13] suggests a need for a larger degree of remodeling, which can be manifested as a more pronounced chromatin fiber unwinding at and/or translocation to the sites of DNA synthesis. Thus, the degree of chromatin compaction may influence the initial steps or dynamics of DNA metabolism in heterochromatin as a result of the involvement of specific molecular remodeling mechanism [14,15]. In DNA repair, the type of remodeling may be also specific to the lesion type and/or pathways involved [16]. In line with that, several studies have reported that induction and repair of DNA damage are affected by the chromatin type [17–20]. Ionizing radiation-induced DNA double strand breaks (DSBs) were demonstrated to occur at lower frequencies in highly condensed heterochromatic regions as compared to euchromatin [18,19,21–23]. On the other hand, DNA damage in euchromatin was demonstrated to be preferentially or faster repaired [18,24–26]. Accordingly, it was suggested that heterochromatin compaction may interfere with DNA damage induction [21] and its repair [27], while dynamic remodeling [28,29] of the chromatin structure represents a prerequisite of efficient DSB repair in heterochromatin [17,30,31]. In line with this hypothesis, transient localized heterochromatin structure relaxation at DSB sites was reported [17,27,30,32]. It was further shown that subsequent chromatin compaction is an important step of DNA damage response [33]. On the other hand, microscopically detectable relocation of damaged DNA out of heterochromatic regions was reported for DSBs induced directly inside heterochromatin [28,29]. A functional significance of such DSB movement to particular nuclear locations for successful completion of DSB repair has been suggested [31,34].

During the course of DNA replication, the nucleotide sequence and epigenetic state of the entire genomic DNA are reliably reproduced through orchestrated duplication of multiple distinct segments of chromosomes – the ‘replicons’ [35]. The existing experimental evidence on the interplay between DNA replication dynamics and chromatin structure has been interpreted within the context of two models. According to one model, the template chromatin fiber moves from inside of a chromatin domain to the proximal replication sites – ‘factories’ [36] – followed by translocation of the replicated chromatin back into compacted chromatin structures, without significant changes in the global large-scale chromatin compaction or shape of these large-scale chromatin fibers [37–39]. Another model proposes sequential *de novo* assembly of replisomes at the individual structural segments of chromatin and a ‘domino’ mode of replication spreading over chromatin fibers within 3D nuclear domains [35,40–45], with only minor local translocation of (nascent) DNA at replication sites [42,46]. Therefore, there are apparently contradictory reports regarding the dynamics of the replication of chromatin domains. According to one line of evidence, chromatin is structurally stable at the level of replication foci [44,47,48] and genome duplication occurs via domino-like activation of individual origins in the underlying chromatin structures [49]. Alternative models postulate translocation of replicating DNA to immobile replication complexes (bodies) at the surface of or adjacent to the larger chromatin structures [38,50] with invariant compaction and shape of chromosome regions (modified ‘replication factory’ model [39]). Thus, for both DNA replication and repair alike, not only localized remodeling and processing of replicating or damaged DNA regions [41,42,49] but also large-scale directional translocations of the DNA to specialized nuclear environments, have been reported [51,52]. Hence, whether assembly of DNA polymerase complexes on heterochromatin structures is associated or not with larger-scale chromatin domain rearrangements remains unclear.

In the present study, we investigate the architecture of constitutive heterochromatin domains in mammalian cells by analyzing DNA replication and repair dynamics in live cells. Our data demonstrate that localized and transient heterochromatin decompaction in the absence of larger-scale translocation takes place and is associated with and possibly triggered by the processive DNA synthesis in DNA replication and damage repair.

## Materials and methods

### Cell culture and transfection

Human HeLa Kyoto cells [53], HeLa Kyoto cells stably expressing cherry-PCNA [49], mouse embryonic fibroblasts (MEFs), C2C12 mouse myoblasts [54] and C2C12 mouse myoblasts stably expressing RFP-PCNA [55] or GFP-PCNA [40] were grown at 37 °C 5% CO_2_ in DMEM supplemented with either 10% (HeLa Kyoto, MEFs) or 20% (C2C12) fetal calf serum and 50 microgram/ml gentamicin. HeLa Kyoto cells stably expressing cherry-PCNA [49] were cultured in the presence of 2.5 micrograms/ml blasticidin. C2C12 mouse myoblasts stably expressing RFP-PCNA [55] were cultured in the presence of 2 micrograms/ml puromycin. Baby hamster kidney (BHK) clone 2 were cultured as described previously [56].

For in vivo DNA labeling, the SiR Hoechst (Spirochrome) dye was added to C2C12 cells stably expressing RFP-PCNA at the final concentration of 500 nanomolar for two hours prior to the live cell microscopy. It was verified that the cells continued proliferation for several cell cycles, suggesting the absence of major cell cycle effect at the dye concentration used.

In fixed cell experiments, cells were grown on 18 mm coverslips and processed as described below prior to microscopy. For live cell microscopy, cells were grown on cover slide dishes (Glass Bottom Dishes, MatTek, 1.5 coverglass thickness), or in 6- or 8-well chambered coverslip dishes (Nunc, Lab-Tek, Chambered Coverglass). Alternatively, cells were grown in p100 or p60 dishes (Nunc) on 40 mm glass coverslips, which were assembled into a FCS2 live cell microscopy chamber (Bioptechs) before live cell microscopy. Trolox (0.5 micromolar) was added to the culture medium in the chamber to minimize photobleaching during microscopy.

Cells were transfected with constructs coding for fluorescently-tagged proteins. In DNA repair experiments, polyethylenimine transfection method was used as previously described [57]. In DNA replication experiments, triple transfection of C2C12 cells was performed using the calcium phosphate transfection method without subsequent glycerol shock [58,59] or using the Neon Transfection System (Thermo Fisher Scientific) according to the manufacturer’s instructions. HeLa Kyoto and BHK cells were transfected using Lipofectamine (Thermo Fisher Scientific) according to the instructions of the manufacturer.

### Expression constructs

The expression constructs used are summarized in **Fig. S1**. All constructs were verified by sequencing, restriction digest analysis, western blot analysis and immunofluorescence analysis.

For DNA replication experiments using C2C12 cells, the following constructs were used: CFP-PCNA [60] (pc922); GFP-PCNA (pc653) [40]; MeCP2-YFP [61] (pc644); MeCP2-miRFP670 (pc3942); and CENPB-DsRed (pc861). MeCP2-miRFP670 codes for full length rat MeCP2 fused to miRFP670 and was generated by replacing GFP in the construct MeCP2-GFP (pc1121) [61] by miRFP670 from pmiRFP670-N1 (pc3379) (gift from Vladislav Verkhusha, Addgene plasmid # 79987) [62] using the restriction enzymes AgeI and MfeI. CENPB-DsRed codes for amino acids 1-169 of human CENPB containing DNA-binding domain and was generated from pCBGS65T (gift from Kevin F. Sullivan) [63] by cutting with HindIII/BglII and cloning into HindIII/BamHI sites of pDsRed1-N1 vector (Clontech).

For DNA replication experiments using HeLa Kyoto cells, the MBD1-GFP expression construct (pc1191) [64] was used.

For DNA replication experiments using BHK cells, the following constructs were used: pSV2EYFP-LACI [56] (pc1021); and pDsRLigase [65,66] (pc822).

For DNA repair experiments, the following constructs were used: mCherry-PCNA [67] (pc1322); MeCP2-GFP [61] (pc1121); MBD1-GFP [64] (pc1191); GFP-XRCC1 [68] (pc1152); and mRFP-XRCC1 [68] (pc1156).

Constitutive heterochromatin was visualized by transfecting cells with constructs encoding for GFP-tagged MBD proteins. In mouse C2C12 cells, chromocenters were identified as MeCP2 enriched regions and in human HeLa cells constitutive heterochromatin of chromosome 1 was identified as MBD1 enriched regions. MeCP2-YFP, miRFP670 or GFP colocalization to chromocenters in mouse cells was confirmed based on 4’,6-diamidino-2-phenylindole (DAPI) staining. Colocalization of MBD1-GFP to heterochromatin regions of chromosomes 1, 9 and 16 of human HeLa cells was tested using fluorescence *in situ* hybridization (see below).

### Detection of processive DNA synthesis

DNA polymerase mediated incorporation of thymidine analogs, 5-bromo-2’-deoxyuridine (BrdU) or 5-ethynyl-2’-deoxyuridine (EdU), was taken as a proxy for the processive DNA synthesis in DNA replication and repair experiments.

To visualize BrdU or EdU incorporation after DNA repair, medium containing 10 micromolar concentration of BrdU or EdU was added to the cells directly before microirradiation. One hour post irradiation, cells were fixed for 10 minutes with 3.7% formaldehyde in phosphate buffered saline solution (PBS) and stained for either BrdU or EdU as described below.

In DNA replication experiments to label nascent DNA in live cells, C2C12 cells expressing GFP-PCNA were scratch loaded [69] with Cy3-dCTP (20 micromolar, Amersham) in complete culture medium and incubated for 20 minutes before replacing the medium. Cells were cultured overnight before performing live cell microscopy.

In double pulse nucleotide labeling experiments, C2C12 cells expressing fluorescent PCNA were first scratch loaded with either Cy3-dCTP or Atto488-dCTP, and incubated in the culture medium for the indicated period of time. EdU at the final concentration of 10 micromolar was then added to the medium and cells were cultured further until fixation with 3.7% formaldehyde in PBS.

### Visualization of incorporated nucleotide and replication protein

For BrdU staining, cells were permeabilized in 0.5% TritonX-100 for 30 minutes and subsequently treated with 10 micrograms per microliter DNaseI in 1x DNaseI buffer (Sigma Aldrich) for 30 minutes at 37 °C. After blocking for 30 minutes with 2% bovine serum albumin in PBS (BSA/PBS) at room temperature, the first antibody (rat anti-BrdU clone BU1/75, Gentaur) was diluted 1:100 in 1% BSA/PBS and incubated for one hour at room temperature. The secondary antibody (Cy5 coupled donkey anti-rat IgG antibody, Jackson ImmunoResearch, #712-175-153) was diluted 1:200 in 1% BSA/PBS and also incubated for one hour at room temperature.

For EdU staining cells were permeabilized in 0.5% TritonX-100 for 20 minutes and ClickIT EdU (Thermo Fisher Scientific) staining solution prepared according to the manufacturer’s instructions was added. Incubation was done for 45 min at room temperature.

For immunofluorescence detection of endogenous replication protein cells were incubated with 10 micromolar EdU for the indicated time, briefly rinsed with PBS and fixed in 4% paraformaldehyde in PBS, followed by incubation for 10 min in ice-cold methanol. Permeabilization was done in 0.5% TritonX-100 in PBS for 20 min. After EdU detection as described above samples were blocked in 2% PBS/BSA and incubated with primary antibodies rabbit anti-PCNA (Epitomics, EPR3821, 1:100 in 1% PBS/BSA) for 2 h at room temperature. Thereafter, cells were washed with PBS supplemented with 0.01% Tween and incubated with secondary antibodies goat anti-rabbit IgG conjugated with Alexa594 (Jackson ImmunoResearch, #111-585-144, 1:400 in 1% PBS/BSA) for 1 h at room temperature. Finally, DNA was counterstained with DAPI (200 ng/ml, Sigma-Aldrich, Germany).

For immunofluorescence detection of the accumulation of endogenous repair proteins following microirradiation, cells were seeded on glass bottom dishes marked with relocation scratches. Fifteen minutes after microirradiation, cells were fixed in 4% paraformaldehyde in PBS, followed by incubation for 10 min in ice-cold methanol. Permeabilization was done in 0.5% TritonX-100 in PBS for 20 min. Samples were blocked in 1% PBS/BSA and primary antibodies were added in 1% PBS/BSA. Mouse anti-PCNA (DABCO, clone PC10, 1:100) and mouse anti-XRCC1 (Abcam clone 33-2-5, 1:200) were added for 2 h at room temperature. For visualization, secondary antibodies donkey anti-mouse IgG conjugated with Cy5 (Jackson ImmunoResearch, #715-175-150, 1:400) were added and incubated for 1 h at room temperature. Finally, DNA was counterstained with DAPI (200 ng/ml, Sigma-Aldrich, Germany) and microirradiated cells were relocated for imaging.

HeLa Kyoto cells expressing mCherry-PCNA were imaged without further signal enhancement. For 3D-SIM applications, the GFP-PCNA signal in C2C12 was increased by incubating the fixed and permeabilized cells with GFP-booster-Atto488 (Chromotek), 1:200 in 0.5% BSA/PBS for 1 h at the room temperature. DNA was counterstained with DAPI (200 ng/ml, Sigma-Aldrich, Germany).

Coverslips were mounted on microscope slides using Vectashield Antifade Medium (Vector Laboratories).

### Immuno-fluorescence in situ hybridization

HeLa cells cultured on glass coverslips were transiently transfected with MBD1-GFP using Lipofectamine (Thermo Fisher Scientific) following the manufacturer’s protocol. Cells were rinsed with PBS and fixed in freshly made 4% paraformaldehyde.

The following human DNA probes were used: repeat specific human DNA probe pUC 1.77 [70] for chromosome 1 (pc3365); alphoid DNA probe pMR9A [71] for the centromeric region 9q12 of chromosome 9 (pc3367); and alphoid DNA probe pHUR-195 [72] for the centromeric region 16q11.2 of chromosome 16 (pc3366). These DNAs were labeled by standard nick translation with Cy5-dUTP (Amersham). DNA was precipitated by adding 2 µl of fish sperm DNA, 15 µl sodium acetate and 150 µl of 100% pure ice-cold ethanol. The precipitation mix was incubated at −75°C for 50 min. The probe was centrifuged at 4°C for 45 min at 13.000 rpm. The pellet was then washed with 70% pure ice-cold ethanol and centrifuged for 30 min. Next, the pellet was air-dried to make sure no ethanol remnants were retained. Then, the probe was resuspended in hybridization solution consisting of 70% formamide, 2x saline sodium citrate buffer, 10% dextran sulfate, pH 7.0 and denatured at 80°C for 5 min.

Cells were permeabilized with 0.25% TritonX-100 in PBS for 10 min, incubated in 0.1 M HCl for 10 min and incubated in blocking solution 4% BSA/PBS for 30 min. Mouse anti-GFP antibody (1:100, Roche, clones 7.1 and 13.1, # 11 814 460 001) was incubated for an hour at room temperature followed by washing and another hour incubation with donkey anti-mouse IgG conjugated with Alexa488 (Jackson ImmunoResearch Laboratories Inc., # 715-545-151, 1:500 in blocking solution). After 15-min post-fixation with 1% paraformaldehyde, the protocol was continued as for FISH. Cells were equilibrated in hybridization solution at 4°C for at least 1 h. Probe and cells were then put together and denatured simultaneously at 75°C for 5 min and hybridized overnight at 37°C. DNA was counterstained with DAPI (200 ng/ml, Sigma-Aldrich) for 10 min and samples were mounted in Vectashield Antifade Medium (Vector Laboratories).

### Microscopy

Imaging and laser microirradiation experiments were performed using a Leica SP5 II confocal laser scanning microscope in a closed live cell microscopy chamber (ACU, Perkin Elmer) at 37 °C in an atmosphere with 5% CO_2_ and 60% humidity. Images were taken with a HCX PL APO 63x/1.49 numerical aperture oil immersion objective. Live cell two-color images were taken simultaneously to increase the temporal resolution of the imaging, whereas fixed four-color images were taken in sequential mode to minimize crosstalk.

Live cell timelapse confocal image Z-stacks were collected with a LSM 510 Meta microscope (Carl Zeiss Microimaging, Inc.), equipped with a 63x/1.4 numerical aperture Plan-Apochromat oil immersion objective at ambient temperature (fixed cells samples), or heated to 37 °C (live cell experiments). In addition, imaging was done on a UltraVIEW VoX spinning disc confocal system (PerkinElmer, UK) mounted on a Nikon Ti microscope (Nikon) equipped with an CFI Plan-Apochromat 60x/1.45 numerical aperture oil immersion objective (pixel size in XY = 111 nm, Z-step 300 = nm) or CFI Plan-Apochromat 100x/1.49 numerical aperture oil immersion objective (pixel size in XY = 67 nm, Z-step 400 = nm).

Super-resolution imaging (3D-SIM) on fixed samples was performed on a DeltaVision OMX V3 system (Applied Precision, GE Healthcare) equipped with a 100 ×/1.40 NA Plan Apo oil immersion objective (Olympus, Tokyo, Japan), Cascade II:512 EMCCD cameras (Photometrics, Tucson, AZ, USA) and 405, 488 and 593 nm diode lasers. Image acquisition and reconstructions were carried out as previously described [73].

Fixed cell samples were also imaged using a Zeiss Axiovert 200 wide-field epifluorescence microscope (pixel size in XY = 104 nm, Z-step = 500 nm) equipped with a Plan-Apochromat 63x/1.4 numerical aperture oil immersion phase contrast objective and a PCO Sensicam QE cooled CCD camera.

### Microirradiation

For laser microirradiation, a preselected spot within the nucleus was microirradiated either with a 405 nm or a 488 nm laser resulting in a total energy of 1 or 3 mJoules, respectively, as described in [68]. Energy output of the lasers was measured with a laser power meter (OPHIR) directly after the objective. Confocal image series of one mid nucleus Z section were recorded either at maximal speed for three images before and after microirradiation and then in 15 s intervals or, for the full time lapse, in 15 s intervals. For evaluation of accumulation, kinetics images were first corrected for cell movement and the mean intensity of the irradiated region was divided by the mean intensity of the whole nucleus (both corrected for background) using ImageJ tools. Maximal accumulation represents the highest ratio from each experiment. In the case of double microirradiation within the same nucleus, the two microirradiation spots were irradiated nearly simultaneously. The latency between the irradiation of the two regions of interest was 40 ms.

### Image processing and analysis

For 3D reconstruction, pictures were taken with a voxel size of 56x56x335.7 nm or 40.4 × 40.4x125.9 nm in C2C12 and HeLa cells, respectively. 3D visualization was performed with the Amira Software (ThermoFisher Scientific) or UCSF Chimera package (Resource for Biocomputing, Visualization, and Informatics at the University of California, San Francisco).

Intensity analysis over one line (line plot) directly through the heterochromatic region was done on one confocal section after filtering with Gaussian blur (radius 1) using ImageJ.

Colocalization analysis of DNA replication (PCNA) and heterochromatin (chromocenter) signals based on Pearson’s coefficient was performed using ‘Manders Coefficients’ ImageJ plug-in [74]. First, a threshold was set to exclude most of the non-focal signal in both channels. Then, the correlation of chromatin and PCNA signals was analyzed for individual chromocenters for each time point, and the analysis results were averaged over the nucleus. Zero-zero pixels were excluded from consideration.

For dynamic analysis of replicating chromocenter parameters (volume, median intensity), Z stacks of the MeCP2 channel were recorded with background intensity close to zero and less than 0.01% of saturated pixels. Chromocenter parameters in individual time points were analyzed using ‘3D object counter’ ImageJ plugin [75]. No preprocessing of image stacks was used. Individual threshold was set for each time point within the plugin interface based on visual inspection of stack to segment nucleoplasm voxels out. Objects less than 20 voxels in size were discarded. For each time point, a measurement table and the measured object map numbered were generated. Object maps for individual time points were inspected to align chromocenter numbering through the course of observation and the data from the individual measurement tables were integrated.

For dynamic analysis of the area of the MBD marked region in DNA damage/repair microirradiation experiments, images were first cropped to the region of interest, convolved with Gaussian of 1.0 pixel radius, thresholded based on the background signal level in the nucleoplasm and subsequently the corresponding segmented area was calculated using ImageJ. The area after irradiation was normalized to the area before irradiation and the relative increase in size was calculated.

Figures were assembled using Adobe Photoshop and Illustrator software. For presentation purposes, linear stretching of the histograms (normalization) was performed independently for each of the channels of the images. Movies were generated by annotating image series and converting them into movie files using ImageJ.

## Results

### Multicolor labeling of replicating heterochromatin domains

Combination of replication labeling with an expression of fluorescently tagged chromatin-binding proteins can be used to investigate changes in the chromatin architecture at nuclear replication sites in all genomic segments. To follow architectural changes of heterochromatin domains in the course of DNA replication, we combined different strategies for *in vivo* labeling of regions of constitutive heterochromatin simultaneously with visualization of replication proteins in S-phase cells.

**Figure 1.**
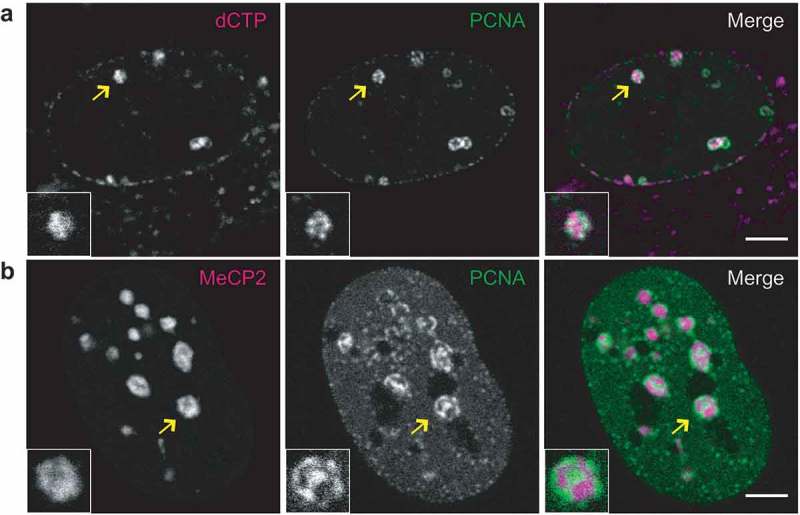
Comparison of replication patterns for differently marked heterochromatin in live cells. (a) Chromatin being replicated in late S-phase was labeled by incorporation of fluorescent nucleotide by incubating C2C12 mouse myoblast cells with Cy3dCTP one cell cycle prior to observation. The replication machinery was visualized by constitutive GFP-PCNA expression. (b) An image of a C2C12 cell transfected with CFP-PCNA to visualize ongoing replication and YFP-MeCP2, which selectively binds to pericentromeric heterochromatin. Chromocenters shown in the magnified inserts are marked by arrows. Scale bars 5 microns.

In the first approach, fluorescent nucleotides were introduced into C2C12 mouse cells expressing a fluorescently tagged component of DNA polymerase complex (GFP-PCNA) [40] using transient mechanical permeabilization of the cell membrane [69]. One day later, we selected for imaging late S-phase cells based on the characteristic-clustered spatial pattern of GFP-PCNA foci. Even without specific staining, the pericentromeric heterochromatin was readily identifiable based on clustered signals from fluorescent nucleotide and replication protein within the chromocenters volume (Figure 1(a)). Similar patterns were observed for chemical and antibody detection of nucleotide analogs and of immuno-detection of endogenous replication proteins (Figure S2A). This excludes the possibility that the localization of the proteins was perturbed by the fusion with fluorescent tags as we have shown in previous publications [40].

In a second strategy, mouse pericentromeric heterochromatin (chromocenters) was specifically labeled by fluorescently tagged methylcytosine binding protein 2 – MeCP2 [61] and combined with the expression of fluorescent PCNA (Figure 1(b)). The advantage of the MeCP2 marking of chromocenters is that it labels the whole volume of the chromocenters, whereas the incorporated nucleotide signal corresponds only to the part of the chromocenters that were replicated during the labeling pulse the day before imaging. Nonetheless, the incorporated nucleotides label directly the genomic DNA, whereas MeCP2 binds to methylated cytosines. Methylated cytosines are enriched in chromocenters [61], which are formed by tandem major satellite repeats of 234-bp long units [76]. In addition, these repeats are AT-rich and, thus, are preferentially bound by the DNA dyes DAPI or Hoechst [77]. We, therefore, seeded a portion of the transfected cells on coverslips to be fixed and directly stained for DNA using DAPI, to highlight these AT-rich condensed DNA regions. Microscopic observation of the preparations demonstrated a general coincidence between fluorescently tagged MeCP2 and DAPI-stained chromocenters as well as localization of PCNA to the inner parts of DAPI-stained chromocenters (Figure S2B). The localization of PCNA to the inner parts of chromocenters was further validated in live cells using the cell permeable DNA dye SiR-Hoechst (Figure S3).

As a third strategy, in human cells, expression of fluorescent methylcytosine binding domain protein 1 – MBD1 – allowed us to visualize heterochromatin blocks that were distributed over pericentromeric regions of several chromosomes, as assayed by fluorescence *in situ* hybridization with probes for different satellite repeat DNA (Figure S4A,B). Replication of the MBD1 labeled heterochromatin in human cells involved localization of the clustered replication signal at the labeled heterochromatin domains (Figure S4A).

As a fourth strategy, in hamster cells, we made use of a previously reported ectopic heterochromatin domain composed of an array of *lacO* sequences detected by the binding of ectopically expressed fluorescent LacI [56] (Figure S4C). Images of the replicating artificial *lacO* array derived heterochromatin domain [56] in rodent cells (Figure S5A,B) frequently showed a pattern of the replication machinery surrounding the densely packed heterochromatic regions, which was in line with previous reports [38].

Contrary to the observations reported for replication sites in early and mid-S-phase [41,48], for all of the four labeling approaches used, signals from nascent DNA and replication proteins never displayed a perfect colocalization with the compact heterochromatin regions. The DNA replication machinery often localized either at the borders of the compacted heterochromatin domains (Figure 1(a), insert, Figure S4, inserts) or in regions of decreased chromatin density within the chromocenters – ‘lacunas’, which were more apparent for bigger chromocenters (Figure 1(b)). Such lacunas, however, did not represent enlarged channels devoid of chromatin, but rather were regions of locally decreased chromatin density containing nascent DNA and replication proteins (PCNA), as confirmed by nucleotide labeling and high-resolution fluorescence microscopy (Figure S6). This implied that replication of various heterochromatin domains involved a step at which replication complexes were localized within the inner parts of heterochromatin domains that was accompanied by decompaction of the underlying heterochromatin.

### Replication dynamics of pericentromeric heterochromatin is associated with sequential localized decompaction of replicating chromatin regions

**Figure 2.**
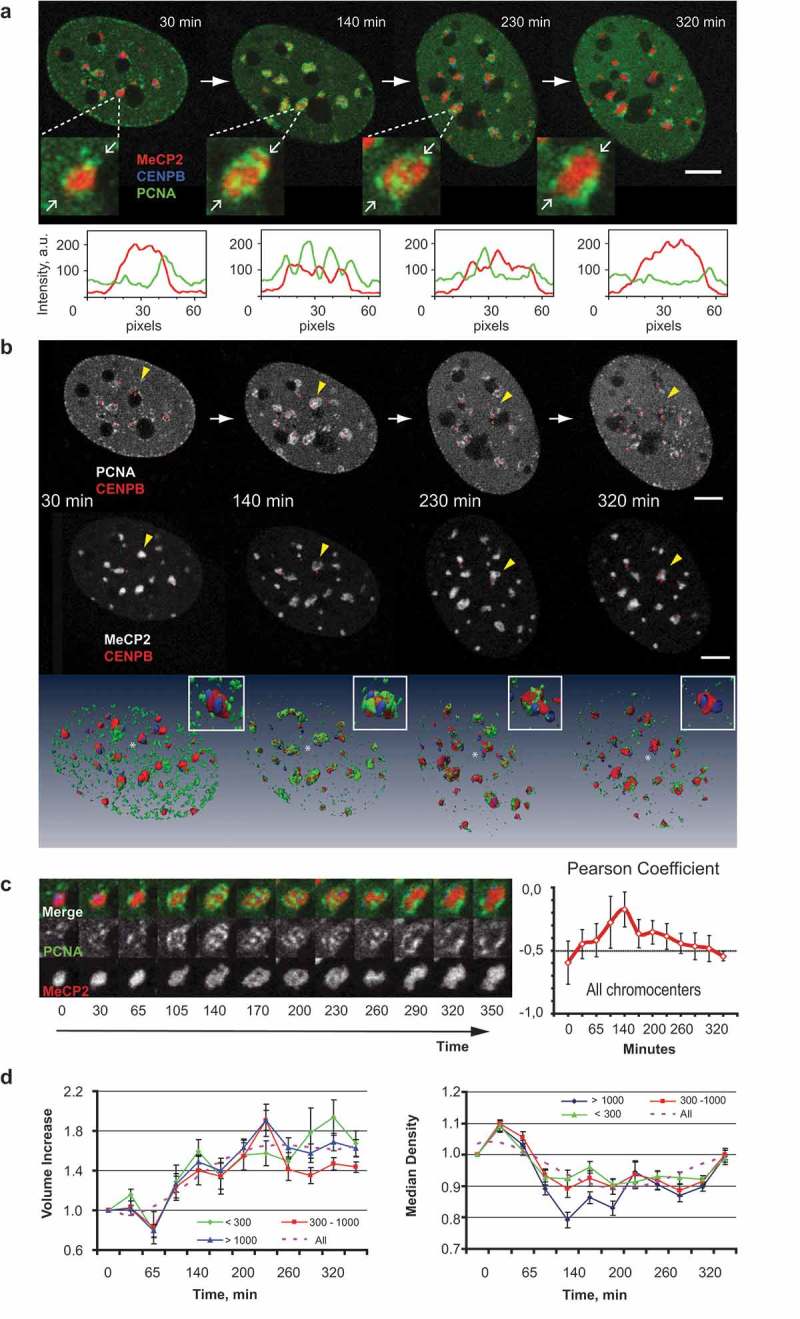
Morphological changes of the pericentromeric heterochromatin domains during DNA replication. (a) Major satellite and minor satellite regions of chromosomes were visualized by expressing MeCP2-YFP (shown in red) and CENPB-DsRed (shown in blue), respectively. Replication sites were marked by CFP-PCNA (shown in green). Replicating cells were followed for several hours starting from the transition from mid to late S-phase. The central sections of Z-stacks of an exemplary cell at four times are shown. The full time lapse as well as full Z-stacks for the cell are presented in Movie 1. Magnified images of the heterochromatin domain marked with yellow arrow are depicted below. The intensity profiles for the line connecting two white arrows in the inserts are shown. Scale bar 5 microns. (b) Central slices and 3D rendering of the C2C12 cell as in (a) showing pair-wise combinations of PCNA/CENPB and MeCP2/CENPB signals at different stages of chromocenter replication (mid to late S-phase). Insert shows the chromocenter marked with yellow arrowhead. Scale bar: 5 microns. (c) An assembly of sections through the central part of an individual chromocenter is shown together with the dynamics of colocalization between MeCP2 and PCNA averaged for all chromocenters in the nucleus measured using Pearson’s coefficient (mean ± standard error of the mean, SEM). Negative values of the Pearson coefficient are indicative of the mutual exclusion between the signals. (d) Dynamics of volume changes (left) and dynamics of reversible decompaction (right, measured as median density of the YFP-MeCP2 signal) for different classes of chromocenters (size <300, n = 11; 300–1000 n = 7; and >1000 voxels n = 9; see also Fig. S9). Average ± SEM for each class and trend line is plotted.

Our initial data on all four DNA/chromatin labeling approaches suggested that replication of compact heterochromatin domains involved positioning of replication complexes both at the domain surface and inside the domain volume. We, then, set out to follow the spatial dynamics of the replicating centromeric and pericentromeric heterochromatin regions in live cells by combining the expression of fluorescently tagged MeCP2 and PCNA with a fluorescent protein component of mouse centromeres (CENPB) (Figure 2(a)).

Despite the essentially continuous nature of genome duplication, the S–phase of the cell cycle can be operationally subdivided into three main sub-periods, each characterized by a unique spatial pattern of replication sites distribution in the nucleus [35]. First, we mapped the time when the replication of chromocenters started with relation to the characteristic S-phase patterns of the replication sites. Initial snapshots of live cells suggested that chromocenter replication started after the characteristic mid-S-phase pattern of replication sites, which is manifested by the presence of perinuclear and perinucleolar chains of PCNA foci (Figure S7A–C). Before and after their replication, chromocenters had a rounded shape. In some cases, PCNA formed rings around chromocenters or a single PCNA focus could be observed in the center of a chromocenter, which suggested a radial symmetry in the organization of pericentromeric chromatin domains (Figure 1(b), Figure S7D). PCNA more often formed rings around smaller chromocenters (Figure 1, Figure 2(a)), and for bigger chromocenters, we observed PCNA signal inside their volume for prolonged periods of time (Figure 2, Figure S7). A more detailed analysis showed that the replication machinery did also colocalize with the inner parts of smaller chromocenters (Figure S8).

We, then, performed a detailed live-cell time-lapse analysis of chromocenter replication on the triple labeled cells, starting from the moment when the overall PCNA distribution pattern corresponded to mid-S-phase (Figure 2(a), compare to similar pattern in Figure S5 with *lacO*/LacI system). It appeared that a mutually exclusive pattern of replication/chromatin signal was a persistent feature in the course of chromocenter replication (Figure 2(a–c)). This observation was further confirmed by quantifying the correlation between heterochromatin and replication derived signals using Pearson’s correlation coefficient (see methods for the analysis details). For the whole span of chromocenter replication, a negative correlation was observed between the signals from both labels, indicative of non-overlapping position of replication machinery and portions of the chromocenters that remained in a compacted state (Figure 2(c)). However, an increase in the value of the coefficient was observed during the replication of the inner parts of chromocenters (Figure 2(c)), which was likely due to the optical overlap of both signals within the heterochromatin domain at the confocal microscopy resolution level. The progression of replication of the pericentromeric heterochromatin followed a particular sequence of positioning of replication sites in relation to the chromocenters. At the beginning of chromocenter replication, PCNA localized around the compacted chromocenter volume, then PCNA foci gradually appeared within the volume of chromocenter, while at the end of the chromocenter replication several PCNA foci remained at the chromocenter surface (Figure 2(a–c)). The observed dynamics was consistent with sequential assembly of the replication foci along the chromosome folding, starting from the surface of chromocenters into the inner parts of them. After replication of the inner parts of a chromocenter has been completed the remaining replication sites again appeared at the surface of the heterochromatin compartment. Interestingly, assembly of PCNA foci inside the chromocenters was accompanied by the formation of lacunas mostly in bigger chromocenters and was associated with a prominent decrease in their median density (Figure 2(c,d)). Like the bigger chromocenters, smaller ones demonstrated transient localization of replication signal within their volume (Figure S8). Live-cell confocal microscopy did not reveal decompaction of the inner parts of small chromocenters or changes in their circularity and the mode of replication of small chromocenters generally resembled that of artificial heterochromatin domains (Figure S5B) where replication colocalized with the inner parts of the domain and of heterochromatin domains in human cells (Figure S4A). However, replication-induced decompaction of the inner parts of smaller chromocenters could be observed at the resolution level of 3D-structured illumination microscopy (Figure S8B). Duplication of DNA in chromocenters eventually lead to an approximately 1.5 fold increase in their volume and a temporary decrease in their compaction (Figure 2(c,d); Figure S9).

**Figure 3.**
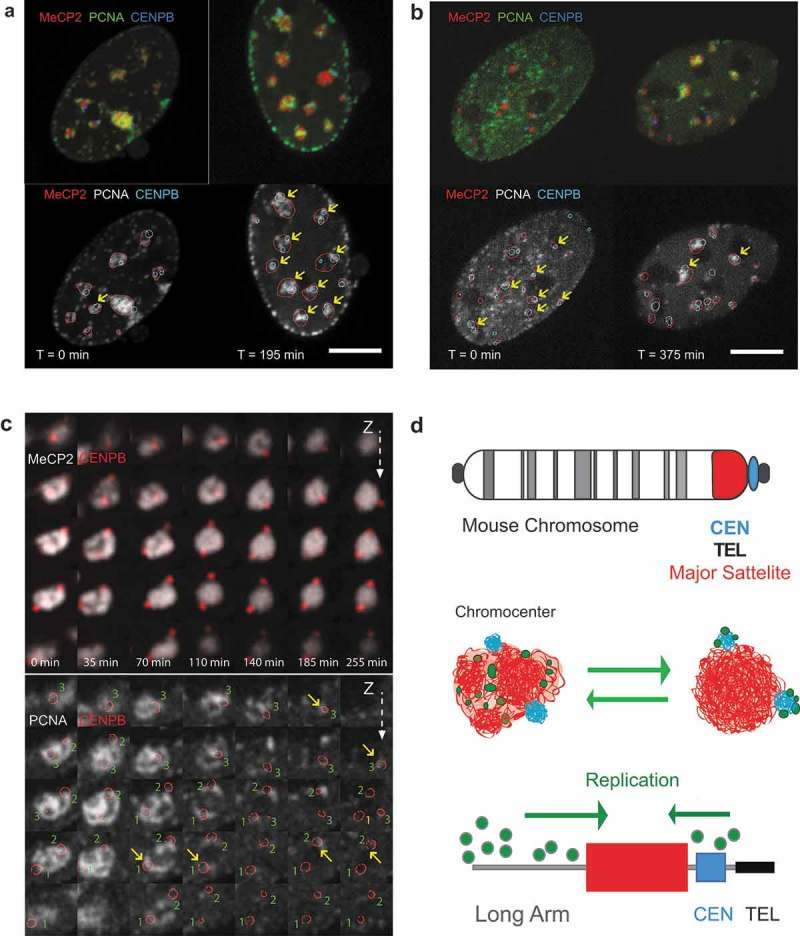
Timing of replication of major and minor satellite domains. (a) Central slices of cells coexpressing fluorescently tagged PCNA, MeCP2, and CENPB (GFP-PCNA, MeCP2-miRFP670, CENPB-DsRed) at the different stages of chromocenter replication: a cell, where replication of minor satellite domains followed replication of major satellite DNA is shown. Chromocenters where CENPB domains colocalized with PCNA are marked with yellow arrows. Scale bar: 5 microns. (b) Central slices of a cell, where replication of most of the minor satellite domains preceded replication of major satellite DNA. Chromocenters where CENPB domains colocalized with PCNA are marked with yellow arrows. Scale bar: 5 microns. (c) Time course of replication of an individual chromocenter consisting of one visible major satellite region and three distinct minor satellite domains, hence, corresponding to three chromosomes. Upper panel shows minor satellites that are marked via CENPB-DsRed binding (shown in red) and MeCP2 marked major satellite regions (shown in grayscale). Bottom panel shows an overlay of PCNA signal (grayscale) and three minor satellite regions marked by red circles. The size of each magnified image is about 3 µm, the Z-spacing between the individual slices is 0.5 µm. Yellow arrows mark when PCNA and CENPB signals of the three minor satellite domains in the chromocenter become partially overlaid indicative of ongoing replication at the corresponding minor satellite domain. While the replication of the major satellite is ongoing at the beginning of the observation (0 min), the three minor satellites show specific colocalization later, at 65 min (1), and at 170–260 min (2,3), indicating that replication of minor satellites takes place after or toward the end of the replication of the major satellite portion of the chromocenter. The full time lapse of the whole cell with the chromocenter framed as well as full Z-stacks are presented in Movie 2. (d) Schematic summary of the observed temporal patterns of replication of major and minor satellite components of chromocenters and suggested model for replication dynamics of the centromere and pericentromeric heterochromatin.

We, next, studied the relative order of replication of adjacent chromosomal segments in the pericentromeric chromatin based on the simultaneous labeling of pericentromeric blocks of major satellite repeats and centromeric blocks of minor satellite repeats in mouse acrocentric chromosomes. Replication of the minor satellite domains was manifested by the formation of PCNA foci adjacent or partially overlapping with CENPB signals (Figure 3(c); Figure S7E). Time-lapse analysis revealed that PCNA foci localized at individual minor satellite signals at different time points, suggesting an asynchronous replication of the minor satellite domains of different chromosomes (Figure 3(a–c)). Notably, replication of centromeric regions mostly occurred after the corresponding chromocenters were replicated (Figure 3(c)). However, in a few cases replication of the minor satellite domains preceded replication of the pericentromeric heterochromatin (Figure 3(b)) [78]. Such sequential order of replication of adjacent chromosomal regions is indicative of 1D spreading (‘domino effect’ [41,44]) of the replication process into the pericentromeric heterochromatin either from the telomeric region or the long arm of the chromosome. This suggests a directional nature of the DNA replication process within heterochromatin blocks of individual chromosomes (Figure 3(d)) as has been previously shown for unique sequences comprised by the reference ‘sequenced genome’ [79].

### Recruitment of DNA damage repair factors inside heterochromatin

In previous work [68], using a combination of 405 nm versus longer wavelength (488 or 561 nm) laser irradiation and control of the illumination energy level, we established laser microirradiation conditions that allow discriminating between processive DNA repair pathways and non-processive short-patch base excision repair. We took advantage of the laser-induced DNA lesions that require processive DNA synthesis as a model system to investigate whether chromatin dynamics in response to the localized DNA damage involves decompaction as we observed for DNA replication.

**Figure 4.**
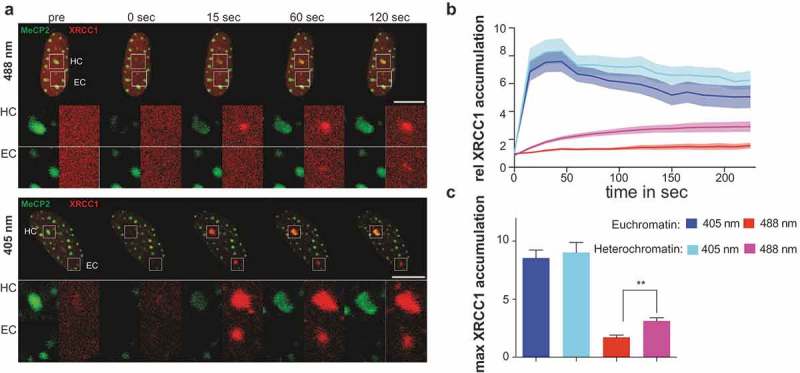
XRCC1 recruitment inside dense constitutive heterochromatic regions. (a) Live-cell imaging plus 488 or 405 nm laser microirradiation of XRCC1 expressing C2C12 cell. Heterochromatin (HC) was visualized by transfection with GFP-tagged MeCP2. Enlarged regions represent sites of irradiation either in heterochromatin (HC) or euchromatin (EC). Full time lapses are shown in Movies 3 and 4. (b) Dynamics of XRCC1 accumulation in C2C12 cells over time shown as mean value ± standard error as shaded region. Full time lapse is shown in Movie 4. (c) Calculation of mean maximal accumulation from curves in (b). Whiskers represent standard error. Statistical test was the Mann Whitney test p < 0.01 * n = between 12 and 19 cells per condition. Scale bars 10 microns.

First, we studied the recruitment of the DNA repair scaffold protein XRCC1 in and outside of heterochromatic regions in relation to the induction of specific DNA repair pathways. The laser beam was directly targeted inside constitutive heterochromatic structures – chromocenters – labeled by GFP-tagged MeCP2 in C2C12 mouse myoblasts (Figure 4). Microirradiation was simultaneously performed outside of chromocenters to investigate possible effects of chromatin compaction on XRCC1 recruitment. In our microirradiation conditions, 3 mJoules 488 nm laser microirradiation resulted in selective activation of short-patch base excision repair whereas 1 mJoule 405 nm laser microirradiation lead to activation of multiple DNA repair pathways, including double-strand break repair (homologous recombination as well as non-homologous end joining) and nucleotide excision repair [68]. Microirradiation with either laser resulted in the accumulation of XRCC1 at irradiated sites (Figure 4(a)). Under both conditions, recruitment of XRCC1 could be observed within the chromocenter volume (Figure 4(a)) as previously reported for heavy ion induced chromatin lesions [29]. Quantification of XRCC1 recruitment revealed a mean increase in accumulation of 9.0 ± 3.4 (mean + standard deviation) after microirradiation with a 405 nm laser and up to 3.1 ± 1.2 fold after microirradiation with 488 nm (Figure 4(b,c)). Notably, XRCC1 accumulation after the 488 nm laser microirradiation was significantly (p < 0.001) stronger inside chromocenters than elsewhere in the nucleus (3.1 ± 1.2 versus 1.7 ± 0.6 fold) (Figure 4(b,c)). This can be caused by the more compact arrangement of DNA in the microirradiated volume and, therefore, a higher level of DNA lesions induced. Moreover, we also controlled for the accumulation of endogenous XRCC1 by microirradiation of living cells that were subsequently fixed and stained for endogenous protein accumulation. As with ectopical expression, we detected XRCC1 accumulation independent of the wavelength (405 nm and 488 nm), although the latter resulted in a more diffuse and weaker accumulation (Figure S10).

We further tested the recruitment of XRCC1 in human cells, where constitutive heterochromatin was visualized using GFP-tagged MBD1 protein as before. Microirradiation with a 405 or 488 nm laser in and outside of these heterochromatic regions resulted in the recruitment and accumulation of XRCC1 (Figure S11A) directly inside heterochromatin. Quantification showed a mean accumulation of 10.7 ± 3.5 and up to 2.5 ± 1.1 fold for 405 and 488 nm microirradiation, respectively, Figure S11B,C). Similar to C2C12 mouse cells, microirradiation with a 488 nm laser inside heterochromatic regions in HeLa cells led to a stronger accumulation of XRCC1 compared to euchromatic regions (2.5 ± 1.1 versus 2.4 ± 1.2 fold), although this difference was not statistically significant (Figure S11B,C).

In summary, these observations demonstrated that, in the selective irradiation conditions, the step of sensing DNA damage was not compromised by the heterochromatin structure or even had a higher efficiency in heterochromatin domains possibly due to higher local density of damage in the compacted chromatin after laser microirradiation.

### DNA repair that involves processive DNA synthesis is associated with local heterochromatin decompaction

**Figure 5.**
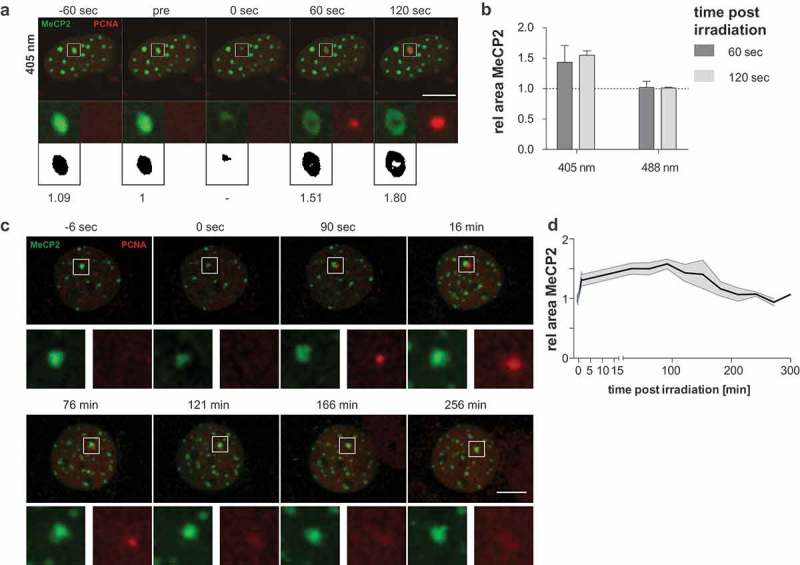
Induction of processive DNA synthesis repair leads to the decompaction of heterochromatin. (a) Live-cell imaging plus 405 nm laser microirradiation of mCherry-PCNA expressing C2C12 cells. Constitutive heterochromatin was visualized by expressing GFP-tagged MeCP2. Full time lapse is shown in Movie 5. Enlarged regions represent sites of irradiation. Binary images: thresholded pictures of heterochromatic GFP-tagged MeCP2 and area size relative to pre-irradiation image. (b) Evaluation of MeCP2 marked area sizes at 60 s and 120 s after irradiation normalized to initial size are shown at the right (mean + standard error). n = between 19 and 22 cells per condition. (c) Recompaction of constitutive heterochromatin was visualized during a time course of 5 h. The decompaction of the heterochromatic compartment occurs within the first seconds to minutes. The compartment remains in the decompacted state for up to 2 h. Then, the recompaction starts as indicated by a decreasing area of the heterochromatic compartment. The recompaction is correlated with the release of PCNA from the microirradiated site (121 min). Scale bar 10 microns. (d) Mean areas of heterochromatic compartments are plotted over time after microirradiation with 405 nm laser and standard error is indicated by the shaded area. n = 10.

Laser microirradiation at 405 nm leads to the activation of several DNA repair pathways, including nucleotide excision repair and double-strand break repair that involve processive DNA synthesis [68]. However, recruitment of XRCC1 directly inside heterochromatic regions may also result from the activation of short-patch base excision repair that represents a non-processive DNA synthesis mechanism. To follow the subsequent steps of DNA repair pathways involving processive DNA repair synthesis, we applied time-lapse microscopy to investigate the recruitment of PCNA, the DNA polymerase processivity factor, to sites of laser microirradiation inside heterochromatin labeled by the methylcytosine binding proteins. We observed PCNA recruitment as soon as 60 s after 405 nm laser microirradiation directly inside constitutive heterochromatic regions (Figure 5(a)). This accumulation was accompanied by decompaction of constitutive heterochromatin in mouse (Figure 5(a,b)) and human (Figure S12) cells. We can rule out that the loss of heterochromatin binding is due to the loss of methylated DNA by replacement with newly synthesized hemimethylated DNA since binding of MBD1 to (hetero)chromatin is independent of the DNA methylation level [60]. Furthermore, the fast recovery of binding of both MBD1 and MeCP2 after laser microirradiation reasons against an effect of photobleaching and lack of binding sites. Irradiation with 488 nm or longer wavelengths did not lead to detectable accumulation of PCNA in non-sensitized cells as published before [68]. Additionally, we controlled for the wavelength-dependent accumulation of endogenous PCNA to the sites of microirradiation by fixing the microirradiated cells and relocation of the same cells after antibody staining against PCNA. Endogenous PCNA only accumulated after microirradiation with 405 nm, but not after 488 nm laser under the microirradiation conditions used (Figure S10).

It was proposed that local chromatin rearrangement with protrusion of the chromatin on the surface of the heterochromatin domain can be a consequence of accumulation of the DNA damage itself [29] or pathway-specific processing of the DNA lesions [34,80]. In that regard, we observed no such apparent decompaction of the damaged regions after inducing non-processive short-patch base excision repair with a 488 nm laser irradiation (Figure 5(b) and S12B). To evaluate whether only processive DNA synthesis repair is associated with localized heterochromatin decompaction, we quantified the area of heterochromatin before and after 405 nm and 488 nm laser microirradiation. 405 nm laser microirradiation led to a 1.4 ± 0.3 (mean + standard deviation) fold area size increase already 60 s after damage induction in C2C12 mouse cells (Figure 5(a,b)), which further increased up to 120-s post-irradiation (1.6 ± 0.3). On the other hand, 488 nm laser microirradiation, which induced only short-patch base excision repair with no processive DNA synthesis involvement [68], did not lead to a significant increase in area size, neither at 60 s nor at 120-s post-irradiation (Figure 5(a,b)). The experiment was repeated in human HeLa cells and produced essentially the same results with size increases of 2.0 ± 0.3 and 2.1 ± 0.3 for 60 s and 120 s respectively (Figure S12B). Next, we measured the re-compaction of the heterochromatic regions following microirradiation with 405 nm laser for 5-h post-irradiation. The increase in the area was very fast in both C2C12 cells (Figure 5(c,d)) as well as in HeLa cells (Figure S12C and D). The maximum was reached within a few minutes after irradiation and the heterochromatic domain remained in the decompacted state for 120 min. Then, in both cases, the heterochromatic regions underwent re-compaction and return to an area comparable to the pre-irradiation size. In both cases, the re-compaction coincided with the release of PCNA from the microirradiated heterochromatin compartment.

### Processive DNA repair synthesis takes place directly in the decompacted parts of heterochromatin domains

**Figure 6.**
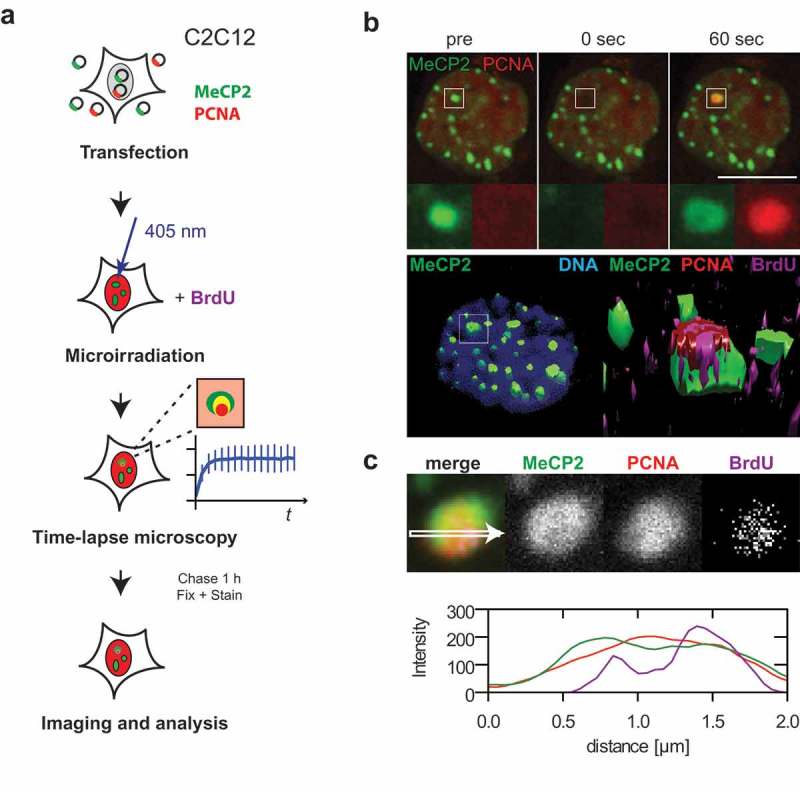
Processive DNA synthesis repair takes place inside dense heterochromatic regions. (a) Schematic overview of the experimental design. (b) Live-cell imaging and 405 nm laser microirradiation of mCherry-PCNA and GFP-tagged MeCP2 expressing C2C12 cells. Full time lapse and Z-stack after fixation and staining are shown in Movie 6. Enlarged regions represent sites of irradiation. 3D reconstructions of irradiated cells. Left: heterochromatic regions and DNA counterstained with DAPI; box represents the site of irradiation. Right: enlarged region of irradiated site; heterochromatic regions display PCNA and BrdU signals inside. (c) Image of one confocal plane from irradiated heterochromatic region and line plot directly through the center. Additional line plots from 10 typical cells are shown in Fig. S13. Scale bar 10 microns.

The accumulation of PCNA led to the question whether, similar to DNA replication, processive DNA synthesis takes place directly in the areas of decompacted heterochromatin or relocation of the damaged DNA out of the heterochromatin domain is required for completion of the repair process [29,34,52,81,82]. To detect repair-related DNA synthesis following damage induction in non-S-phase cells, modified thymidine analogs (BrdU or EdU) were added to the cells directly before 405 nm laser microirradiation, as shown in a schematic overview in Figure 6(a). To compensate for the low intensity of DNA repair synthesis and allow reliable detection of the DNA synthesis, the irradiated cells were incubated with the thymidine analogs for 1 h before fixation and staining for BrdU or EdU. Line intensity profiles through one confocal microscopy plane as well as 3D reconstruction of the cells revealed colocalized PCNA and BrdU signals inside the heterochromatin compartment (Figure 6(b,c)). This was observed in all cells where we microirradiated heterochromatic compartments and stained for BrdU incorporation. A gallery for 10 exemplary cells is shown in Figure S13. The above observations were also verified in human cells (Figure S14) where BrdU incorporation following microirradiation with 405 nm lasers was also found inside MBD1 labeled heterochromatic domains. The BrdU/EdU incorporation was always colocalized with the PCNA signal. We did not observe significant differences between the EdU or BrdU incorporation (Figure S14C). A sample gallery of different cells is shown in Figure S14D.

## Discussion

Chromatin structure has been shown to influence many aspects of cellular metabolism under normal and pathological conditions [83]. Despite that basic principles of higher-order chromatin organization are more than ever the subject of vivid debates [6], the existence of active nuclear compartments having decreased chromatin density where most of the nuclear processes occur, has been generally accepted [84–86]. Quantitatively, heterochromatin occupies most of the metazoan genome [87] and has its unique set of epigenetic and structural properties, including a relatively compact structure [11,88]. It has been documented that the structural organization of the nuclear heterochromatin domains may influence the nuclear processes that involve these segments of the genome [18,19,60,89]. In particular, previous observations suggested that unlike euchromatin, compact heterochromatin structure prevents efficient DNA metabolism in the inner parts of the heterochromatin domains, and DNA needs to translocate to the domain surface to be repaired, or replicated [31,37,38]. In contrast to that, ordered (sequential) ‘reading’ of preexisting chromatin structures by the DNA replication machinery in the course of DNA replication was also reported [44,46,47,49] and modeled [45,90] at the domain and single replicon levels.

In the present study, we followed the DNA replication and repair dynamics in constitutive heterochromatin of live mouse cells as the main experimental system and validated our observations in human cells. Chromocenters represent microscopically visible spatially clustered pericentromeric sequences from one or several chromosomes. Mouse chromosomes are mostly acrocentric and contain large pericentromeric arrays of gamma-satellite DNA repeats (major satellites) adjacent to ~600 Kbp blocks of 120-bp alpha-satellite repeats (minor satellites) of the centromeric region, followed by the telomere [50,91,92]. Highly CpG-methylated major satellite regions recruit MeCP2 protein [93], a member of the MBD family of proteins [94], whereas minor satellites are bound by the CENPB protein [95]. Therefore, to analyze the dynamics of DNA replication in mouse pericentromeric heterochromatin, we expressed fluorescent MeCP2, DNA polymerase processivity factor (PCNA) and CENPB in mouse cells. Additional labels for heterochromatin included MBD1-bound satellite domains in human cells and an artificial *lacO* array heterochromatin segment in hamster cells [56]. To compare and extend our study to another DNA synthesis-based process, we made use of previously verified laser microirradiation conditions to selectively activate DNA damage repair pathways that also involve processive DNA synthesis in mouse or human cells [68].

Our live cell-derived data demonstrate that in both, DNA replication and DNA repair, processive DNA synthesis takes place directly inside the heterochromatin domains and is associated with local heterochromatin decompaction at the synthesis sites as evidenced by local decrease in signal from chromatin-bound proteins and DNA dyes, both in fixed and live cells (Figure 2, Figures S2,S3). In the case of DNA replication, this effect was most pronounced for the inner parts of relatively large chromocenters that were also used as primary irradiation targets in the DNA repair experiments. In smaller chromocenters and in other examples of heterochromatin domains, replication complexes were detected and the DNA was synthesized also inside the heterochromatin domain volume, but the degree of decompaction of the inner parts of the domain could not be visualized with live-cell confocal microscopy. However, localization of DNA replication to decompacted areas of small chromocenters could be detected by super resolution 3D-SIM (Figure S8) as well as in fixed cells even at wide-field microscopic resolution (Figure S8C). This suggests that the spatial and/or temporal scale of heterochromatin domain decompaction and/or rearrangement may depend on the size of the domain and/or its inner structure. Localization of PCNA foci proximal to minor satellite DNA (Figure 3(c), Figure S7E) and minor shifts between the nucleotide and PCNA signals observed in high-resolution microscopy images may be explained by small-scale translocations of the chromatin fiber, e.g., at the scale of DNA loops. The observed dynamics of the replication foci at the minor satellite domains (Figure 3(b,c)), may be also used to estimate the scale of the chromatin domains at which redistribution of the nascent DNA occurs. Minor satellite blocks in mouse chromosomes have a size of about 600 Kbp [50], which corresponds well with the size of topologically associated [96] and replication [97] domains.

**Figure 7.**
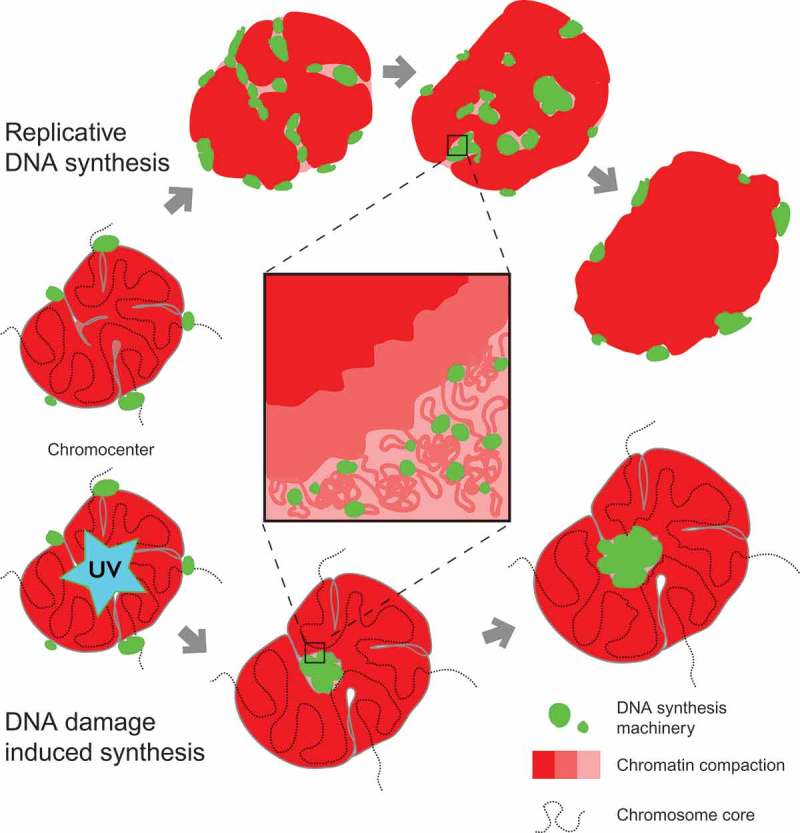
A model for chromatin dynamics during replication and repair in mouse chromocenters. The upper part shows the sequential steps of a heterochromatin domain (chromocenter) replication. Chromatin is duplicated by the sequential assembly of DNA replication complexes along the chromosome fiber fold. Replication of chromatin in the inner parts of the chromocenter occurs locally, which leads to decompaction of the chromatin at the sites of DNA synthesis. Translocation/rearrangements of the template and nascent DNA take place at the subdomain scales, which may correspond to chromatin loops/replicons. The bottom part shows chromatin decompaction at the site of laser-induced DNA damage. Unlike DNA replication, damaged sites that are engaged in DNA repair induced processive DNA synthesis are randomly affected by the laser beam and may localize at significant distances along the chromosome. Simultaneous processing of the damaged sites leads to the formation of ‘DNA synthesis compartment’ within the chromocenter and the corresponding increase in its overall volume.

Our data show that localization of DNA replication complexes inside the chromocenter volume is a transient state, before and after which DNA replication machinery localizes at the surface of the heterochromatin domains (Figure 2). Such ordered 3D dynamics of chromocenter replication is compatible both with previous observations [38] of the duplication bodies at the surface of heterochromatin domains and the suggested principle of the genetic continuity of the replication foci [41,44]. Therefore, the surface mode of chromocenter replication observed in previous studies [38,50] represented a snapshot of the nuclear replication foci dynamics in pericentromeric heterochromatin domains, corresponding to the moment when replication complexes assemble at the interface between eu- and heterochromatic nuclear areas. Our data show that this takes place before the replication machinery assembles within the heterochromatin domain volume inducing localized decompaction of the inner parts of the domain (Figure 7). Eventually, the replication machinery ‘emerges’ from the inside of the heterochromatin domain and replication complexes again appear at the surface of the domain.

Unlike in the recently proposed ‘adjacent replication factory’ model of DNA replication [39] proposed based on analyzing ectopic DNA arrays, our data suggest that replication is associated with local changes in compaction and/or arrangement of the heterochromatin domains that is associated with the assembly of DNA replication complexes. The previous fixed-cell data [38,50], on the other hand, could not distinguish between chromatin movement following its replication and assembly of new replication complexes in the proximity of chromatin segments that have already been replicated.

Replication of major satellite domains spanned from mid to late S-phase [76]. In accord with the previous reports [50], we also observed an asynchronous manner of replication of pericentromeric and centromeric heterochromatin domains with minor satellite domains generally replicated after duplication of bulk pericentromeric major satellite. However, in some cells, most of centromeric DNA replicated earlier than the corresponding pericentromeric heterochromatin, which corresponds to previous microscopic observations in mouse cells [78] and next-generation sequencing analysis of centromere replication timing in human cell lines [98]. The differences in relative order of replication of centromeric DNA and pericentromeric heterochromatin are indicative of the large-scale plasticity of the DNA replication program in individual cells. These observations further suggest that the domino principle of progression of genome duplication [41,46] is in effect at multiple domain scales. Accordingly, it may be speculated that chromatin is decompacted at the macrodomain level as a result of gradual spreading of DNA replication along the chromosome, with the elementary chromatin domains being processed in sequential order (Figure 7).

We could not reveal chromatin decompaction before the accumulation of PCNA signal as a marker of ongoing processive DNA synthesis. We cannot exclude that the temporal resolution of imaging was not sufficient to record fast decompaction events that precede the accumulation of the processivity factors. At the same time, the function of such preliminary decompaction would be unclear. It has been shown that heterochromatin is readily accessible for proteins up to 50 kDa [99,100] and individual components of replication machinery as well as DNA repair complexes are, thus, able to penetrate the inner parts of the heterochromatin domains [29,34]. In line with that, our analysis of DNA repair dynamics after laser-induced DNA lesions demonstrated that recruitment of DNA repair factors occurs inside and outside of heterochromatin independently of the type of damage-induced (Figure 4, Figure S11). Accumulation of the DNA repair scaffolding protein – XRCC1 – after selective activation of the short-patch base excision repair pathway was significantly higher in constitutive heterochromatin than in euchromatin as was also previously reported for double-strand break repair [31]. The reason for elevated XRCC1 accumulation in heterochromatin might simply be due to denser compaction of DNA and, consequently, more potential DNA molecules targeted by the laser beam. This also proves that compact heterochromatin structure does not prevent molecules of DNA repair factors from access to various types of DNA lesions including double-strand breaks, base damage and single-strand breaks that are induced directly inside dense heterochromatic regions.

It is tempting to speculate that processive DNA synthesis itself may lead to major scale rearrangement of higher-order chromatin structures. For example, this can be a consequence of local changes in physical characteristics of the chromatin fiber [101] and/or by a topological stress induced by moving replication forks [102]. Redistribution of the signal from the newly synthesized DNA may in turn result from pulling DNA through individual replisomes at the replicon/loop level. In accordance with the above hypothesis, the signal from the nucleotides incorporated after inducing processive DNA repair synthesis spread over the damaged chromocenters (Figure 6, Figure S14).

Once many processive DNA synthesis complexes are assembled on the DNA, they may impose cooperative topological stress on higher-order chromatin structures (Figure 7). The structure of heterochromatin may be further destabilized by local nucleosome modifications and action of architectural proteins and chromatin-remodeling complexes leading to dynamic disassembly of chromatin domains [14,89,103]. Local transient changes in physical parameters of the chromatin as a result of action of DNA replication or DNA repair proteins may lead to liquid-liquid phase separation and formation of a ‘processive’ heterochromatin subcompartment at the sites of DNA synthesis where DNA metabolism takes place [101,104].

Within the above hypothesis, the overall level of local domain decompaction should correlate with the DNA synthesis intensity. Accordingly, the observed differences in the scale and duration of replication-induced decompaction of larger and smaller chromocenters may be a consequence of different local density of replisomes (Figures 1(b),2(b), Figure S8). This can also explain the apparent contradiction between our data on processive DNA synthesis associated decompaction of heterochromatin domains after laser irradiation, and the lack of major architectural changes described in case of heavy-ion induced [29], ionizing radiation-induced [81] or enzymatic cleavage derived DSBs [34]. Our verified laser irradiation conditions [68] lead to activation, inter alia, of long patch base excision repair pathway with patches of approximately 25–30 nucleotides being synthesized. Given the limitations on the minimal laser spot size in the range of 200 nm – 500 nm, the damaged area contained multiple patches that were repaired/synthesized at the same time and the overall DNA synthesis intensity can approach the one in DNA replication. This is unlikely the case for the above-mentioned damage induction methods.

Whether DNA synthesis associated chromatin decompaction is a specific characteristic of heterochromatin domains or represent a general principle of dynamic chromatin organization is still open. Further studies including high-resolution live-cell experiments will be required to analyze DNA synthesis associated chromatin rearrangements at the scale of individual chromatin domains and within euchromatin, where DNA replication and repair associated chromatin decompaction and/rearrangement are likely to be less pronounced.
