# Supplementary material for: Processive DNA synthesis is associated with localized decompaction of constitutive heterochromatin at the sites of DNA replication and repair
Source: Nucleus. 2019 Nov 19;10(1):231–53. doi: 10.1080/19491034.2019.1688932 (PMC6949026; doi:10.1080/19491034.2019.1688932)
Supplement: Supplemental Material [file kncl-10-01-1688932-s001.zip › Supplementary information/KNCL_2019_0009_R_clean_supplemental_figure_movie_legends.docx]

**Processive DNA synthesis is associated with localized decompaction of constitutive heterochromatin at the sites of DNA replication and repair**

Vadim O. Chagin^1,2^, Britta Reinhart^1^, Annette Becker^1^, Oliver Mortusewicz^3^, K. Laurence Jost^1^, Alexander Rapp^1^, Heinrich Leonhardt^3^ and M. Cristina Cardoso^1^

^1^Cell Biology & Epigenetics, Department of Biology, Technische Universität Darmstadt, Germany

^2^ Institute of Cytology, Russian Academy of Sciences, St. Petersburg, Russia

^3^ Department of Biology II, LMU Munich, Germany

**Correspondence to:**

M. Cristina Cardoso

Department of Biology

Technische Universität Darmstadt

Schnittspahnstrasse 10

64287 Darmstadt

Germany

cardoso@bio.tu-darmstadt.de

Phone: +49-6151-16-21882

Fax: +49-6151-16-21880

**Present address:** Oliver Mortusewicz, Science for Life Laboratory, Department of Oncology-Pathology, Karolinska Institutet, S-171 21 Stockholm, Sweden

**Keywords**: DNA replication, DNA repair, pericentromeric heterochromatin, chromocenters, chromatin compaction, processive DNA synthesis, genome architecture, PCNA

**Figure S1. Relevant parts of expression constructs used in this study.**

Schematic representation of relevant features of the expression plasmids used in this study. Plasmid collection number (pc…), structure of the fusion proteins and, on the right hand side, the corresponding reference are shown. Drawings are not scaled.

**Figure S2. Colocalization of MeCP2 and PCNA to DAPI stained chromocenters.**

(**A**) Localization of the endogenous PCNA and nascent DNA to the inner parts of DAPI stained chromocenters. (**B**) Central slice of a fixed cell transiently transfected with GFP-PCNA and MeCP2-miRFP670 showing the correspondence of MeCP2- and DAPI-visualized chromocenters. The inserts show two-fold magnified chromocenters marked with arrows. Scale bar: 5 microns.

**Figure S3. In vivo DNA staining of replicating chromocenters.**

Central optical section of a live C2C12 cell stably expressing mRFP-PCNA incubated with SiR-Hoechst. Scale bar 5 microns.

**Figure S4. Visualization of replicating heterochromatin domains.**

(**A**) Replication of constitutive heterochromatin visualized by transient expression of MBD1-GFP protein in HeLa cells stably expressing mCherry-PCNA. Magnified regions are marked by arrows. (**B**) Colocalization of MBD1-GFP construct with heterochromatin regions of chromosomes 1, 9 and 16 visualized by immuno-FISH in HeLa cells (see methods for details). (**C**) Replication of heterologous heterochromatin domain visualized by coexpression of YFP-LacI and DsRed-Ligase fusions in BHK cells containing a genomically integrated array of *lacO* sequences. Scale bars 5 microns.

**Figure S5. Dynamics of replication of a heterologous heterochromatin domain in hamster cells.**

(**A**) Central section of the BHK cell and individual magnified images of Z-sections of the LacI/LacO domain (green) at the time of its replication (red). (**B**) Dynamics of colocalization between replication and the LacI/*lacO* domain. Scale bar 5 microns.

**Figure S6.** **3D-SIM image of GFP-PCNA expressing mouse C2C12 cell loaded with a fluorescent nucleotide.**

Cell was scratch loaded with Atto590-dUTP and fixed 20 minutes later. A magnified image of channel overlay for the framed chromocenter is shown. Scale bar 5 microns.

**Figure S7. Dynamics of colocalization of PCNA and (peri)centromeric heterochromatin.**

Characteristic patterns of replication sites (**A**) before, (**B**) during and (**C**) after chromocenter replication. Line plots for the chromocenter (visualized with MeCP2) marked with arrowheads are presented. (**D**) Images showing a radial symmetry between chromocenters and replication sites. Centromeric (CENPB) regions are shown in the same cell together with pericentromeric (MeCP2) regions. (**E**) Relative localization of the replication sites (PCNA in gray scale), major satellite regions (MeCP2 in blue) and minor satellite signals (visualized with CENPB in red) during centromere replication. Scale bar 5 microns.

**Figure S8. Localization of PCNA signal during replication of small chromocenters.**

(**A**) Confocal section and orthogonal views of CFP-PCNA in a C2C12 cell co-transfected with fluorescently tagged MeCP2. (**B**) 3D-SIM images of replicating chromocenters. (**C**) An epifluorescence microscopy image of a MEF cell incubated with EdU for 10 minutes, fixed with formaldehyde and stained for EdU and DNA. Scale bar 5 microns.

**Figure S9. Dynamic changes in volume and mean intensity of chromocenters in the course of DNA replication.**

Combined volume of all chromocenters and mean intensity per chromocenter voxel for a C2C12 cell transfected with GFP-PCNA, MeCP2-miRFP670 and CENPB-DsRed. Measurements for individual time points and polynom-fitted trendline are shown.

**Figure S10. Accumulation of endogenous DNA repair proteins after laser microirradiation.**

Detection of endogenous proteins after microirradiation using 405 and 488 nm lasers. Live cell microirradiation was performed using proteins tagged with fluorescent proteins (two images at the top, before and 60 seconds post microirradiation). Fifteen minutes post irradiation cells were fixed and the accumulation of endogenous proteins was tested using antibody staining. The same cells were relocated and imaged (lower four images). While irradiation with 405 nm leads to the recruitment of both endogenous PCNA and XRCC1, irradiation with 488 nm does not lead to a detectable accumulation of PCNA, but still to the accumulation of XRCC1. Scale bar: 5 µm.

**Figure S11. XRCC1 gets recruited inside dense constitutive heterochromatic regions in the nuclei of human cells.**

(**A**) Live cell imaging plus 488 or 405 nm laser microirradiation of XRCC1 expressing HeLa cell. Heterochromatin (HC) was visualized by transfection with GFP-tagged MBD1 protein (MBD1). Full time lapses are shown in Movies 7 and 8. Enlarged regions represent sites of irradiation either in heterochromatin (HC) or euchromatin (EC). (**B**) Dynamics of XRCC1 accumulation in HeLa cells over time as mean value + standard error as shaded areas. (**C**) Calculation of mean maximal accumulation from curves in (**B**). Whiskers represent standard error. n = between 10 and 15 cells per condition. Scale bars 10 microns.

**Figure S12. Induction of processive DNA synthesis repair leads to decompaction of heterochromatin.**

(**A**) Live cell imaging plus 405 nm laser microirradiation of mCherry-PCNA expressing HeLa Kyoto cells. Constitutive heterochromatin was visualized by expressing GFP-tagged MBD1. Full time lapse is shown in Movie 9. Enlarged regions represent sites of irradiation. Binary images: thresholded pictures of heterochromatic GFP-tagged MBD1 and area size relative to pre-irradiation image. (**B**) Evaluation of MBD1 marked area size 60 seconds and 120 seconds after irradiation normalized to initial size are shown at the right. Whiskers represent standard deviation. n = between 14 and 16 cells per condition. Scale bar 10 microns. (**C**) Recompaction of constitutive heterochromatin was visualized during a time course of five hours. The decompaction of the heterochromatic compartment occurs within the first seconds to minutes. The compartment remains in the decompacted state for up to two hours. Then, the recompaction starts as indicated by a decreasing area of the heterochromatic compartment. The recompaction is correlated with the release of PCNA from the microirradiated site (121-166 min). (**D**) Mean areas of heterochromatic compartments are plotted over time after microirradiation with 405 nm laser, standard error is indicated by the shaded area. n= 11.

**Figure S13. Sample gallery of ten typical line profiles showing BrdU incorporation in microirradiated heterochromatin compartments.**

Line profile analysis from 10 cells similar to Fig. 6C. Chromocenters labeled by MBD1-GFP in C2C12 cells were microirradiated with 405 nm laser, incubated in BrdU containing medium for one hour and subsequently fixed. After antibody detection the cells were relocated and the line profiles for MBD1, PCNA and BrdU incorporation are depicted across the irradiated chromocenter. BrdU incorporation is spatially correlated to the PCNA location and, in most cases, located inside the chromocenter.

**Figure S14.** **Processive DNA synthesis repair takes place inside dense heterochromatic regions.**

(**A**) Schematic overview of experimental design. (**B)** Live cell imaging and 405 nm laser microirradiation of PCNA and GFP-tagged MBD1 protein expressing HeLa cells. Full time lapse is shown in Movie 10. Enlarged regions represent sites of irradiation. 3D reconstruction of irradiated cells. Left: heterochromatic regions and DNA counterstain with DAPI; box represents site of irradiation. Right: enlarged region of irradiated site; heterochromatic regions display PCNA and BrdU signals inside. (**C**) Image of one confocal plane from an irradiated heterochromatic region and line plot directly through the center. (D) Additional sample line profiles from four typical cells. Identical to (**C**). (**E**) Same experiment in HeLa cells incubated with EdU. Scale bar 10 microns.

**Movie 1** Time lapse showing replication of chromocenters in a C2C12 cell transiently transfected with CFP-PCNA, MeCP2-YFP and CENPB-DsRed.

**Movie 2**. Time lapse showing replication of major and minor satellite subcompartments of chromocenters in a C2C12 cell transiently transfected with CFP-PCNA, MeCP2-YFP and CENPB-DsRed.

**Movie 3** Time lapse of 488 nm microirradiation of MeCP2-GFP labeled heterochromatin in a C2C12 cell expressing mRFP-XRCC1.

**Movie 4** Time lapse of 405 nm microirradiation of MeCP2-GFP labeled heterochromatin in a C2C12 cell expressing mRFP-XRCC1.

**Movie 5** Time lapse of 405 nm microirradiation of MeCP2-GFP labeled heterochromatin in a C2C12 cell expressing Cherry-PCNA.

**Movie 6** Time lapse of 405 nm microirradiation of MeCP2-GFP labeled heterochromatin in a C2C12 cell expressing Cherry-PCNA together with animated Z-stack showing nucleotide incorporation at the site of the irradiation.

**Movie 7** Time lapse of 488 nm microirradiation of MBD1-GFP labeled heterochromatin in a HeLa Kyoto cell expressing mRFP-XRCC1.

**Movie 8** Time lapse of XRCC1 accumulation following 405 nm microirradiation of MBD1-GFP labeled heterochromatin in a HeLa Kyoto cell expressing mRFP-XRCC1.

**Movie 9** Time lapse of 405 nm microirradiation of MBD1-GFP labeled heterochromatin in a Cherry-PCNA expressing HeLa Kyoto cell.

**Movie 10** Time lapse of 405 nm microirradiation of MBD1-GFP labeled heterochromatin in a Cherry-PCNA expressing HeLa Kyoto cell together with animated Z-stack showing nucleotide incorporation at the site of the irradiation.
